# Supplementary material for: Bacterial contributions to delignification and lignocellulose degradation in forest soils with metagenomic and quantitative stable isotope probing
Source: ISME J. 2018 Sep 26;13(2):413–29. doi: 10.1038/s41396-018-0279-6 (PMC6331573; doi:10.1038/s41396-018-0279-6)

Figure S1. Reaction scheme for the synthesis of ^{13}C ring-labeled coniferyl alcohol which was subsequently polymerized into lignin with an average of fourteen coniferyl alcohols. Carbon-13 isotopes are denoted in light blue. Consult the Supplementary Methods for a complete description of synthesis reactions.

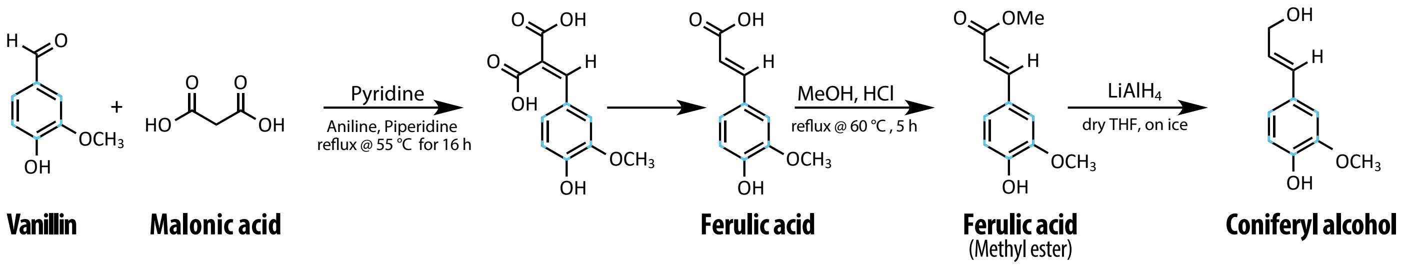

Figure S2. Evidence for the improved assembly of metagenomes derived from ^{13}C-enriched DNA in soil microcosms amended with ^{13}C-labeled (blue) or unlabeled (pink) cellulose or lignin. Statistically supported differences between paired labeled and unlabeled treatments (t-test; $p < 0.01$) are designated with an asterix (*). Statistical testing could not be performed for cellulose with single ^{12}C-libraries. When composited, ^{12}C-libraries cellulose libraries had significantly lower assembly than ^{13}C-libraries in organic (Wilcoxon; $p < 0.003$) and mineral layer soils (Wilcoxon; $p < 0.001$).

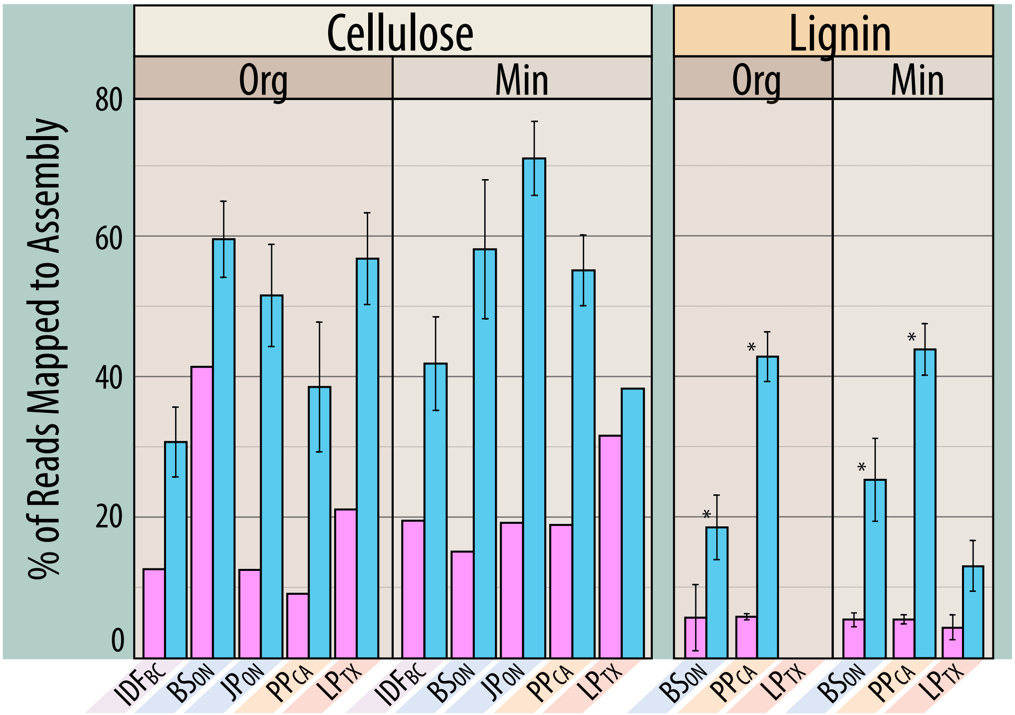

Figure S3. The relative abundance of prominent lignocellulolytic members of Burkholderiales based on differential abundance between ^{12}C- and ^{13}C-libraries. Plots are organized in a hierarchical structure displaying family and genus for all active taxa within each group. Error bars correspond to one standard error of the mean. Significant (Tukey HSD; $p_{adj} < 0.05$) pairwise differences are grouped by lettering.

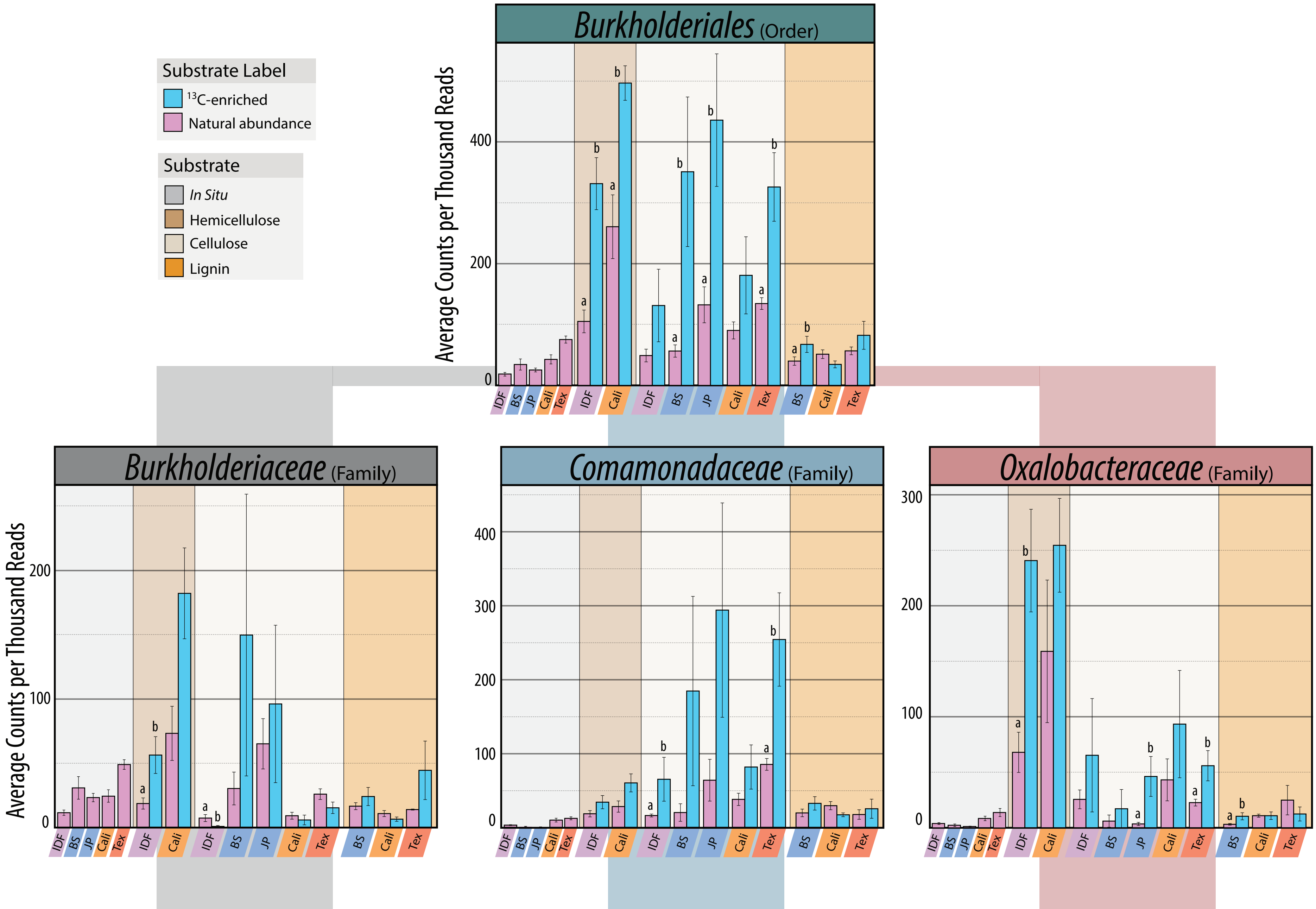

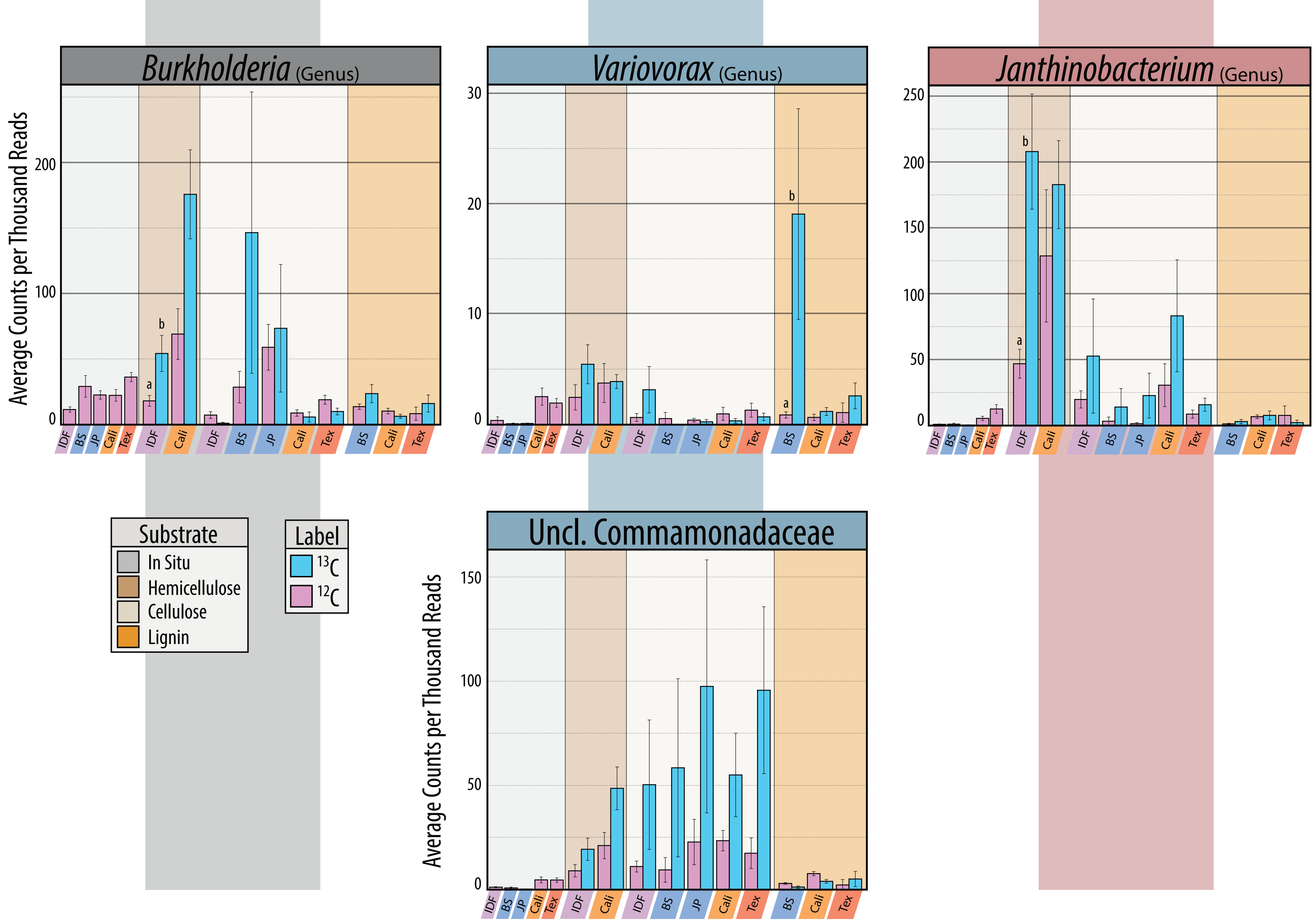

Figure S3 continued...

Figure S4. The relative abundance of all OTUs classified to the genera of Caulobacteraceae. Differences in hemicellulolytic, cellulolytic and ligno-lytic can be observed among genera and unclassified Caulobacteraceae sequences based on differential abundance between ^{12}C- and ^{13}C-libraries. Error bars correspond to one standard error of the mean. Significant (Tukey HSD; $p_{adj} < 0.05$) pairwise differences are grouped by lettering.

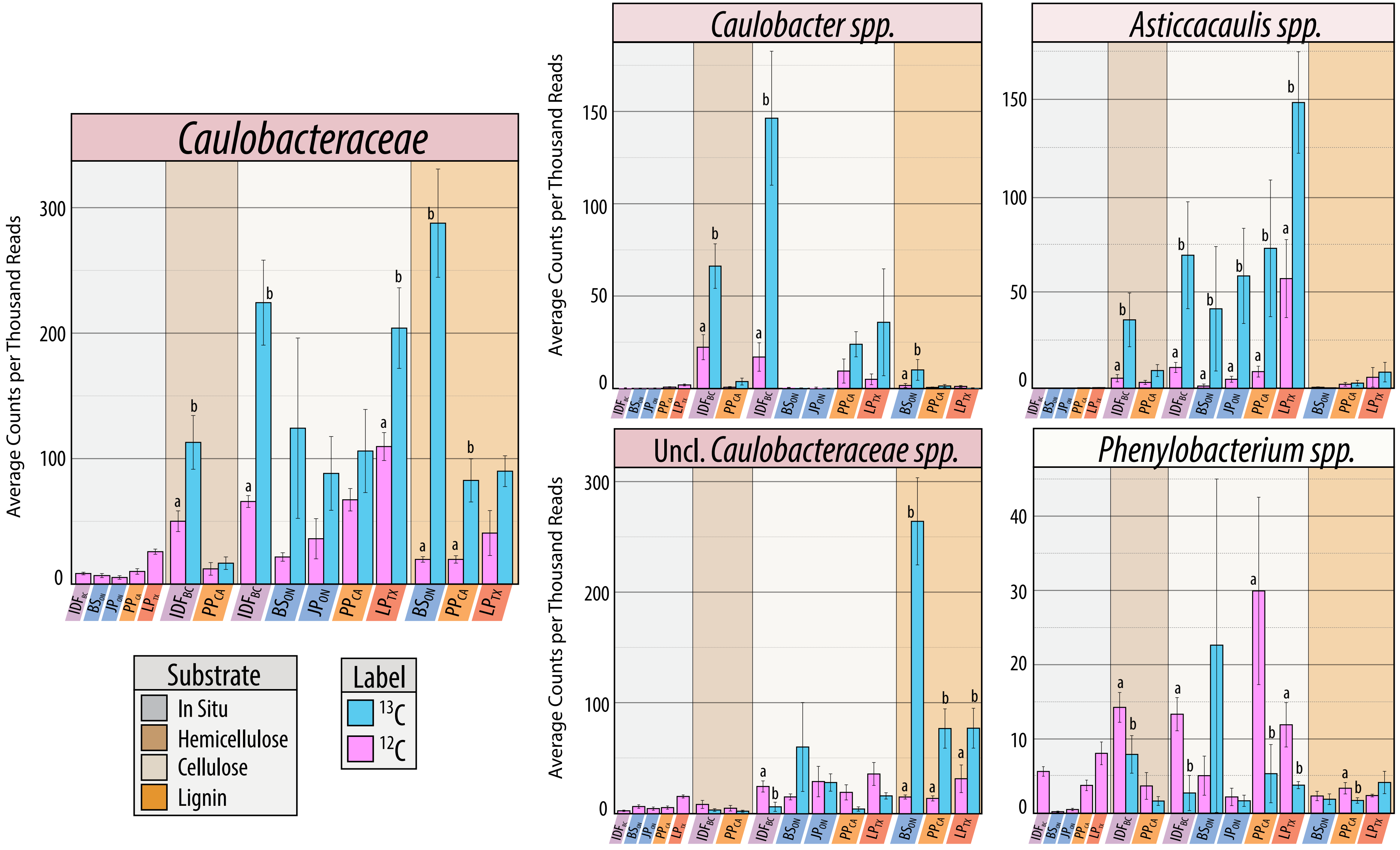

Figure S5. Maximum parsimony tree showing phylogenetic distribution of lignocellulolytic OTUs within the family Caulobacteraceae. Clades are named based on SILVA tree and custom names were assigned to putatively functional clades. Branches were coloured to indicate membership to broader clades inset at the top left. The closest cultured represtatives were included where possible. Clades from this study are comprised of multiple OTUs with the total number provided in parentheses.

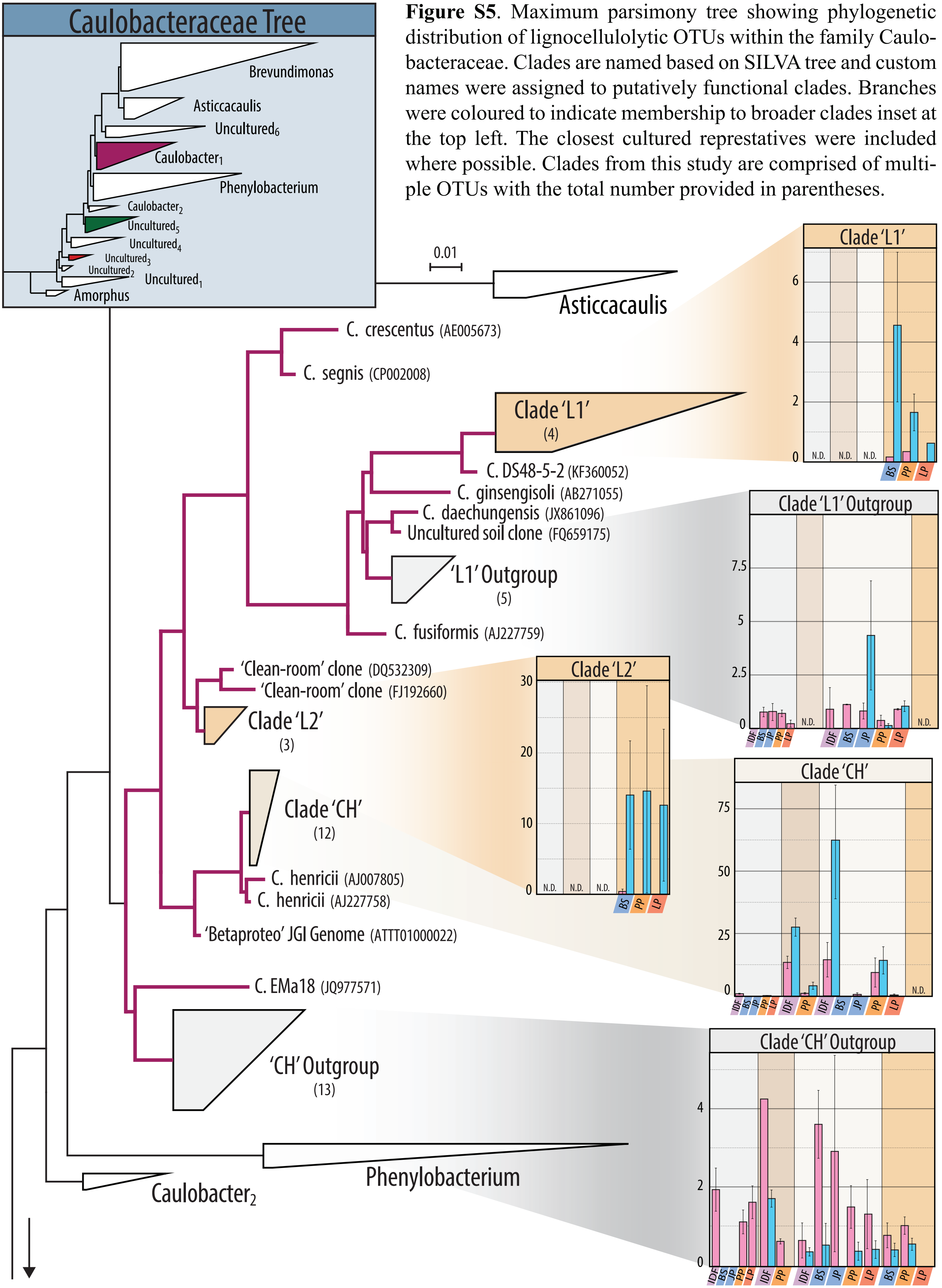

Figure S5 continued...

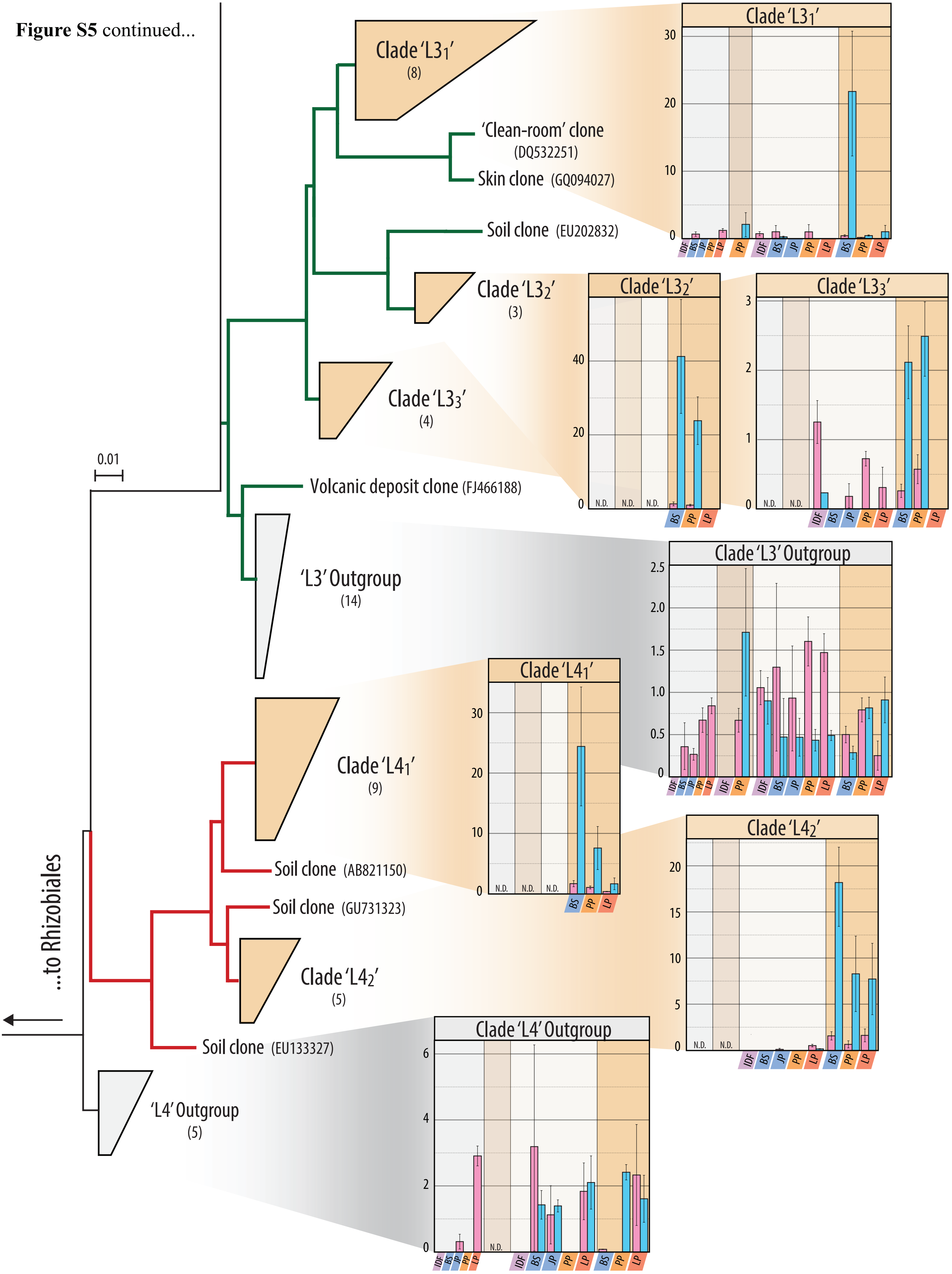

Figure S6. Abundances of OTUs enriched in both lignin and cellulose ^{13}C-pyrotag libraries within the same region. Plots are titled with the lowest supported taxonomic classification and include representative read names. Where two OTUs were combined, both independently exhibited the trend in multiple substrate use. The unclassified Caulobacteraceae OTU belongs to the clade 'LH3_1' presented in Supplementary Figure 3b.

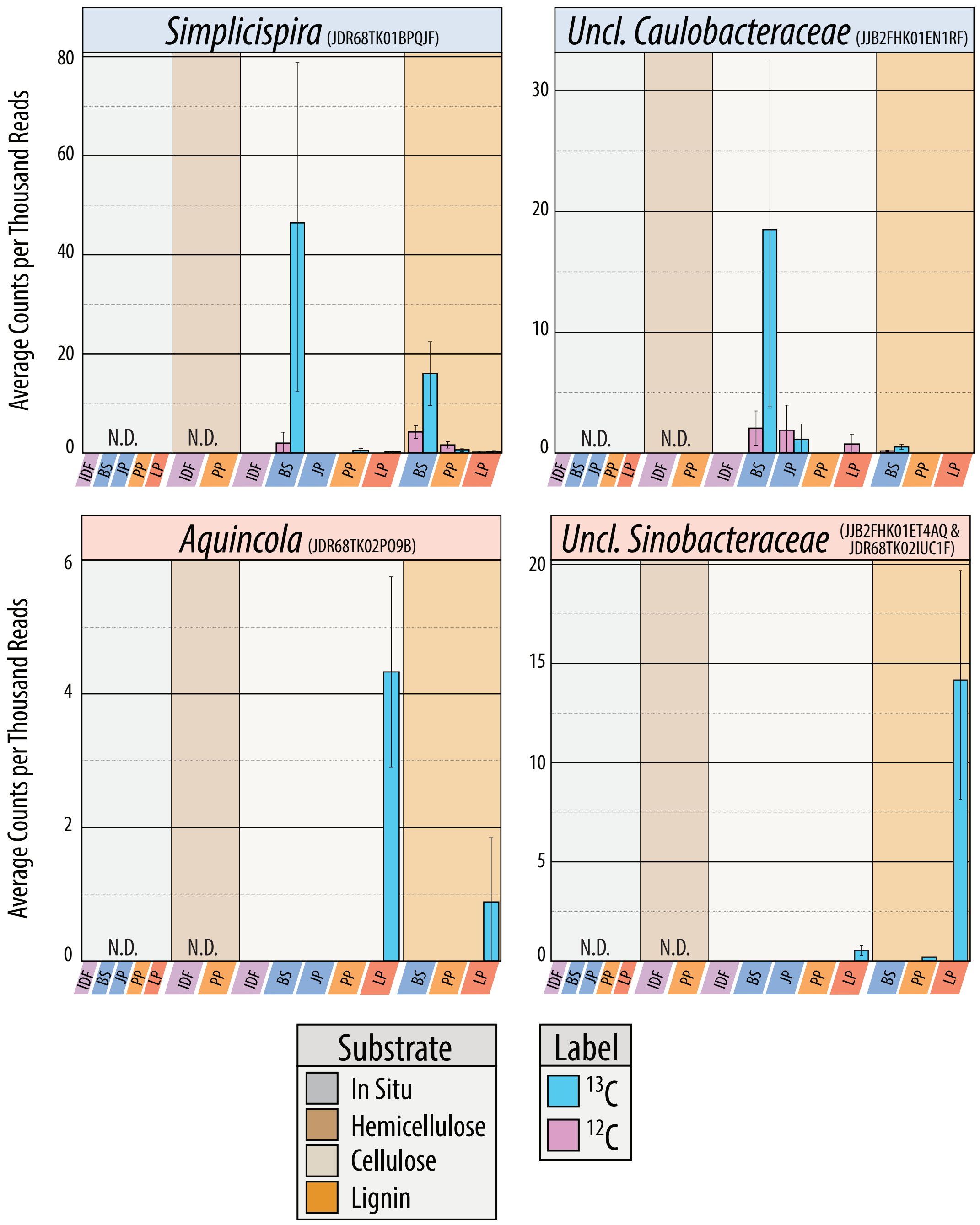

Figure S7. Relative abundances of cellulolytic taxa unique to IDF_{BC}. The relative abundances of bacteria are based on 16S rRNA gene pyrotag libraries and fung on LCA classification of unassembled shotgun metagenomes. Error bars correspond to one standard error of the mean.

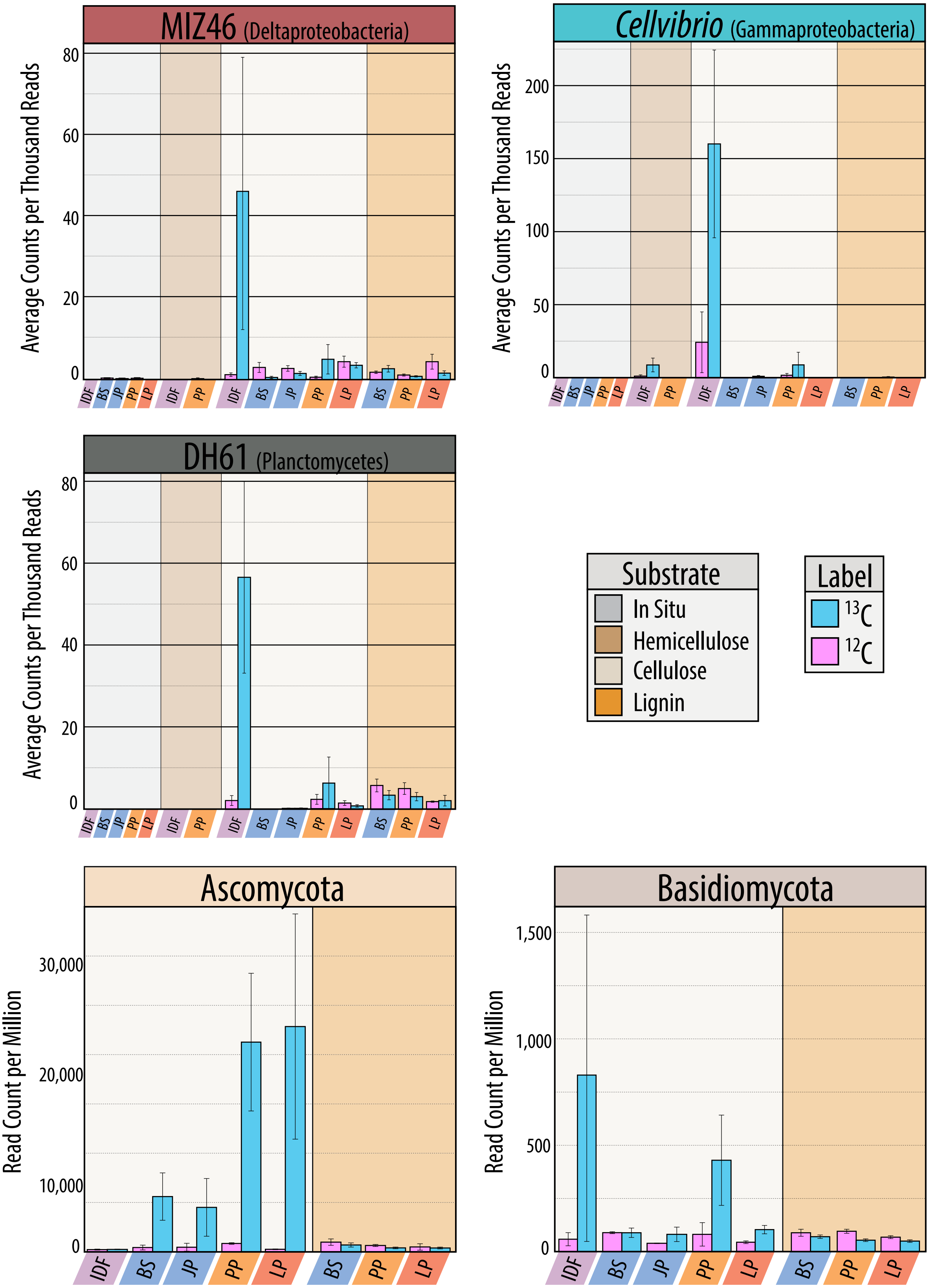

Figure S8. The average delta-^{13}C enrichment of PLFA markers for fungi, gram-positive bacteria and gram-negative bacteria for all three substrate amendments.

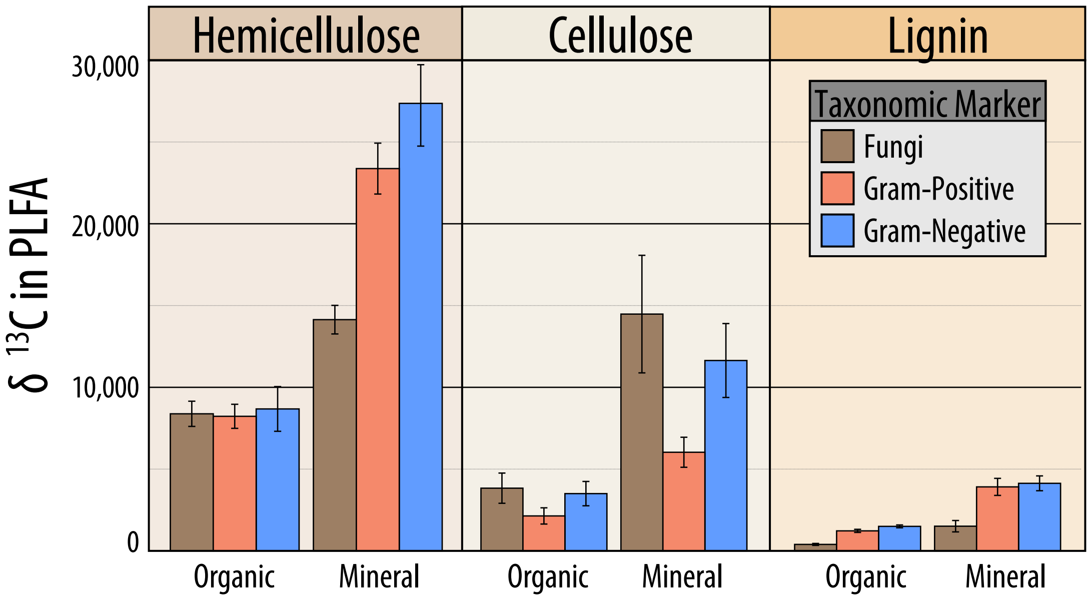

Figure S9. Taxonomic classification of catabolic genes in CAZy clusters in 13C-metagenome assemblies, including (A) genes encoding lignin-modifying enzymes, (B) clusters containing a CBM and an endoglucanase, and (C) all endoglucanase-containing gene families. All taxa represented by a single gene were not displayed. Taxonomic classifications were assigned based on LCA and functional annotation based on top blast hit (e-value < 10^{-5}) to CAZy database or positive match to custom hidden Markov models.

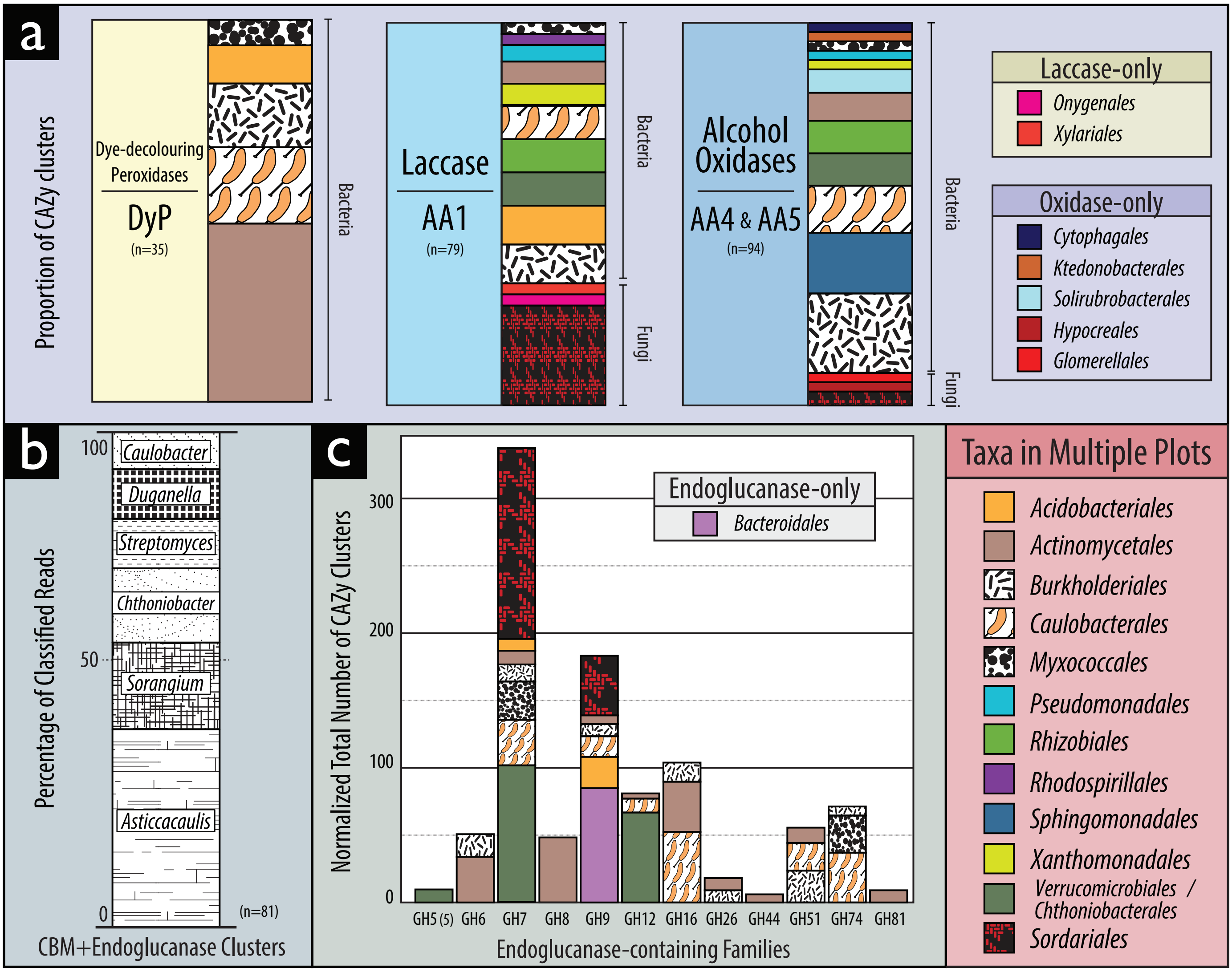

Supplement: Supplementary file 1 — Supplementary Figures [file 41396_2018_279_MOESM1_ESM.pdf]
