# Supplementary material for: Bacterial contributions to delignification and lignocellulose degradation in forest soils with metagenomic and quantitative stable isotope probing
Source: ISME J. 2018 Sep 26;13(2):413–29. doi: 10.1038/s41396-018-0279-6 (PMC6331573; doi:10.1038/s41396-018-0279-6)
Supplement: Supplementary file 10 — Supplementary Methods [file 41396_2018_279_MOESM10_ESM.docx]

*Synthesis of Coniferyl Alcohol from ring-labeled or unlabeled Vanillin*

The complete reaction scheme is presented in Figure S1.

**Step 1**: Aldol condensation of vanillin in the presence of malonic acid

Malonic acid and vanillin (1:1 molar ratio) were condensed in the presence of catalytic amounts of piperidine and aniline (~6 drops) in pyridine. The reaction was performed in a 3-neck flask (100 ml) containing 10 ml pyridine, 2.5 g vanillin and 2 g malonic acid. The mixture was refluxed at 55 °C for ~16 h. The refluxed mixture was removed from the flask and 60 ml chloroform was added. The resulting ferulic acid was extracted (six times) as precipitate using acidic water (pH ~2). To this was added residual ferulic acid extracted using ethyl acetate. The identity and purity of the ferulic acid were determined by TLC (1:1 ethyl acetate/hexane) in comparison to a standard.

**Step 2:** Esterification of ferulic acid

Ferulic acid (2.5 g) was mixed with 10 ml methanol and ~50 μl conc. HCl in a 3-neck flask. The mixture was refluxed at 65-70 °C for 5-6 h. The progress of the reaction was followed on TLC and additional HCl was added as needed. The methyl ferulate was extracted from the completed reaction using ethyl acetate and the sample was dried under vacuum. The final yield was ~95% of the starting ferulic acid.

**Step 3:** *Reduction with Lithium Aluminium Hydride (LiAlH)*

Methyl ferulate was reduced using LiAlH in a 2-neck flask, which was set up with an add funnel under positive nitrogen pressure. The flask was pre-weighed with 0.732 g of LiAlH, to which 60 ml of freshly distilled THF was added slowly while stirring. Methyl ferulate (~2.5 g) was mixed with 14 ml of freshly distilled, dry THF and placed in the add funnel. The flask was kept on ice and ester was added very slowly dropwise. Towards the end, some additional THF was used to rinse the add funnel. The solution was stirred at room temperature for additional 2 h and then cooled to 0 °C. At this point, 12 ml ethyl acetate was added drop wise, followed by 70 ml of 2 N HCl. These were added slowly due to the reactivity of the residual LiAlH. The produced coniferyl alcohol was extracted using ethyl acetate, dried under vacuum, and purified using silica chromatography. The purity of coniferyl alcohol was tested using TLC and Gas-Chromatography coupled Mass Spectrometry.

*Preparation, purification and purity assessment of bacterial cellulose and DHP lignin*

Bacterial cellulose was produced by growing *Gluconacetobacter xylinus* str. KCCM 10100 with ^13^C-labeled glucose (99 atom % 13C, Cambridge Isotope Laboratories, MA, USA) in Yamanaka media under conditions outlined in Ruka *et al.,* (2012). To ensure media was highly oxygenated, a requirement for the production of cellulose, cultures were grown in a 200-mL volume in a much larger 2 L Erlenmeyer flask with sponge tops tightly covered with aluminum foil to retard evaporation. Cellulose was purified according to Dunford (2011) but with longer boiling times (4 h) and three repetitions of boiling in 1% sodium hydroxide. Following purification and soaking the cellulose in a mildly acidic solution, contamination from DNA and protein was not detectable by spectrophotometry (A_260_ and A_280_). The carbohydrate composition of bacterial cellulose was assayed by HPLC after dissolving in strong sulfuring acid. The custom made bacterial cellulose was 100% glucose in comparison to the commercially available plant-derived ^13^C-cellulose from IsoLife: 58% glucose + 4.4% lignin + remainder hemicellulose. . DHP lignin was synthesized from ring-labeled or unlabeled coniferyl alcohol using horseradish peroxidase as described Kirk and Brunow (1988). DHP lignin was washed twice in distilled water to remove residual unpolymerized coniferyl alcohol and its purity verified by HPLC. The molar mass of DHP was determined by Gel Permeation Chromatography using THF as solvent and a standard curve prepared using polystyrene standards.

*Analysis of Phospholipid Fatty Acids*

PLFAs were extracted from 0.75 g (organic) or 1.0 g (mineral) dry wt soil according to Bligh and Dyer (1959) and ^13^C-content was analyzed using ion ratio mass spectrometry (UBC Stable Isotope Facility) ported with gas chromatography as detailed in Churchland *et al.,* (2013) with the following exceptions: (i) methyl undecanoate (c11:0) was used for the internal standard, and (ii) quantitation was based on an average of three serial dilutions of undecanoate, nonadecanoate (c19:0), and methyl cis-13-docosenoate (c22:1ω9). Peak identification was based on retention time compared against two reference standards: bacterial acid methyl-ester standard (47080-0; Sigma–Aldrich, St. Louis) and a 37-Component fatty acid methyl-ester mix (47885-U; Sigma–Aldrich, St. Louis). Unidentifiable enriched peaks, termed “unidentified fatty acids” (UFA), were included in analysis if they met the following conditions: i) detection in > 3 samples, ii) average δ ^13^C > +50 ‰ and iii) confirmed as long-chain alkane methyl esters by GC-MS using the identical instrumentation and method as in Leckie *et al.,* (2004). Taxonomic affiliations of specific PLFAs were assigned according to Högberg *et al.,* (2013), with c18:1ω9 and c18:3ω6 added as additional fungal PLFAs (Ruess and Chamberlain, 2010). All comparisons between taxonomic groups were performed using values normalized to total PLFA content. All SIP-PLFA raw data was processed identically in R using scripts that can be found at: <https://github.com/roli-wilhelm>.

*Overview of Sequencing Libraries*

Over 260 amplicon libraries, averaging 8,400 reads per sample, provided grounds of comparing unincubated field samples (n = 94) and SIP-microcosm experiments for hemicellulose (n = 47), cellulose (n = 62) and lignin (n = 62). Whole shotgun metagenomic and PLFA data were prepared only for cellulose and lignin, amounting to 33 and 48 PLFA profiles, respectively, and 38 and 46 shotgun metagenomic libraries with an average of 86 million and 56 million quality filtered 100 bp reads per library. Approximately half of all data was derived from ^12^C-control microcosms and an overview of all datasets can be found in Table S2.

*Separation and Recovery of ^13^C-enriched DNA from Soil DNA Extracts*

DNA was extracted from 0.5 g soil with the manufacturer’s recommended protocol for the FastDNA™ Spin Kit for Soil (MPBio, Santa Ana, CA). The mass and the atom % ^13^C of DNA extracts were measured with UHPLC-MS-MS according to Wilhelm *et al.* (2014). ^13^C-enriched DNA was recovered by density gradient ultracentrifugation according to methods outlined in Neufeld *et al.* (2007) with the following modifications: i) 20 fractions of ~ 300 uL were collected, ii) the amount of DNA applied to the column was standardized according to the degree of ^13^C enrichment, resulting in between 6 – 10 µg of DNA used per sample and iii) the resuspension of DNA from the cesium chloride solution was performed more efficiently and effectively using Amicon Ultra-0.5 mL filters (EMD Millipore, MA, USA) in place of PEG_6000_. Heavy fractions (1.727-1.735 g/mL, typically fractions 1-7) were pooled (max 500 µL) and concentrated in the filter columns by centrifugation for 3 min at 14,000 rcf. Samples were then washed three times with PCR-grade water.

*Characterization of Carbohydrate-Active Enzyme (CAZy) Content*

The complete CAZy database (Cantarel *et al.,* 2008; [www.cazy.org](http://www.cazy.org)) was downloaded and formatted into a DIAMOND blastx searchable database (accessed 2014-05-06, for Chapter 3, and again on 2015-08-19 for Chapter 4). The database was downloaded with a script implemented in Metapathways 2.5 (Konwar *et al.,* 2015) which is publicly available at https://github.com/nielshanson/CAZy_utils. Metagenomic reads were annotated by CAZy family based on the top hit of blastx searches with minimum e-value of 10^-5^, as described in Cardenas *et al.,* (2015). The following glycosyl hydrolase (GH) families encode enzymes with endoglucanase activity: GH5, 6, 7, 8, 9, 12, 26, 44, 45, 48, 51, 61, 74, 81 and 131 (Yagüe *et al.*, 1990; Hasper et al., 2002; Petersen *et al.,* 2009; Harris *et al.,* 2010; Vlasenko *et al.,* 2010; Warner *et al.*, 2011; Lafond et al., 2012; Boyce *et al.*, 2015; Brumm *et al.*, 2015). The following GH families encode enzymes with xylanase activity: GH10, 11, 30, 67, 115 and 120 (Zifcakova *et al.* 2017). The following AA families encoded enzyme with ligninase activity: AA1, 2, 3, 4, 5, 6 and 12 (Levasseur *et al.*, 2013). A custom python script (available at <https://github.com/Roli-Wilhelm/CAZY_Clusters>) was used to identification of clusters of CAZymes which first identified putative CAZyme sequences by a) blasting against the CAZy database, b) using ‘hmmscan’ (HMMER v. 3.1b1; Eddy, 2011) using hidden Markov models for CAZymes provided by dbCAN (Yin *et al.,* 2012) and custom hidden Markov models for aryl and vanillyl alcohol oxidases, laccases, dye decolorizing peroxidases (Dyp2), and versatile peroxidases (Erick Cardenas, unpublished, also available in previously mentioned github repo). The script then identifies groups of three or more CAZymes on assembled scaffolds and recovered these sequences. This script implemented gene-predicting software Prodigal (Hyatt *et al.,* 2010; v. 2.6.2). Clusters containing carbohydrate binding modules were analyzed for activity related to cellulose degradation to improve the likelihood that encoded genes were involved in similar functions. ORFs encoding genes from the β-ketoadipate pathway (KEGG Module M00568), vanA (K03862) and vanB (K03863) were identified by BLAST searched against the KEGG database (downloaded June 13^th^, 2016).

**References**

Boyce A, Walsh G (2015) Characterisation of a novel thermostable endoglucanase from Alicyclobacillus vulcanalis of potential application in bioethanol production. *Appl Microbiol Biotechnol*, **99**: 7515-7525.

Brumm PJ, Hermanson S, Gowda K, Xie D, Mead DA (2015) *Clostridium thermocellum* Cel5L-cloning and characterization of a new, thermostable GH5 cellulase. *Int J Biochem Res Rev*, **6**: 62.

Harris PV, Welner D, McFarland KC, Re E, Navarro Poulsen JC, Brown K *et al.* (2010) Stimulation of lignocellulosic biomass hydrolysis by proteins of glycoside hydrolase family 61: structure and function of a large, enigmatic family. *Biochem*, **49**: 3305-3316.

Hasper AA, Dekkers E, van Mil M, van de Vondervoort PJI, de Graaff LH (2002) EglC, a new endoglucanase from *Aspergillus niger* with major activity towards xyloglucan. *Appl Environ Microbiol*, **68**: 1556-1560.

Lafond M, Navarro D, Haon M, Couturier M, Berrin J-G (2012) Characterization of a broad-specificity β-Glucanase acting on β-(1,3)-, β-(1,4)-, and β-(1,6)-Glucans that defines a new glycoside hydrolase family. *Appl Environ Microbiol*, **78**: 8540-8546.

Petersen L, Ardèvol A, Rovira C, Reilly PJ (2009) Mechanism of cellulose hydrolysis by inverting GH8 endoglucanases: a QM/MM metadynamics study. *J Phys Chem B*, **113**: 7331-7339.

Vlasenko E, Schülein M, Cherry J, Xu F (2010) Substrate specificity of family 5, 6, 7, 9, 12, and 45 endoglucanases. *Biores Technol*, **101**: 2405-2411.

Warner CD, Go RM, García-Salinas C, Ford C, Reilly PJ (2011) Kinetic characterization of a glycoside hydrolase family 44 xyloglucanase/endoglucanase from *Ruminococcus flavefaciens* FD-1. *Enzyme Microb Technol*, **48**: 27-32.

Yagüe E, Béguin P, Aubert J-P (1990) Nucleotide sequence and deletion analysis of the cellulase-encoding gene *celH* of *Clostridium thermocellum*. Gene 89: 61-67.
